# Scurvy: Its Cause

**Published:** 1864-09

**Authors:** W. S. Oliver

**Affiliations:** Assistant Surgeon 4th Battalion 60th Rifles.


### ART. III.—*Scurvy: Its Cause.* By W. S. OLIVER, M. D., Assistant Surgeon 4th Battalion 60th Rifles.

For the following remarks respecting the cause of scurvy, I do not desire to credit myself with complete originality, nor are my assertions substantiated on my part by pathological or practical research; but so confident am I that the true cause of scurvy entirely depends on a deficiency of protein compounds, both animal and vagetable, in the food used by sufferers from that disease, that I cannot refrain from giving publicity to my views, theoretical though they be, with a hope that at some future period they may be practically verified.

My reason for making this assumption are the facts collected from the symptoms of the disease, the action of the supposed scorbutics and antiscorbutics, and the treatment adopted owing to the ill-formed theories as to its cause.

The languor and despondency, the indisposition to bodily action, the fatigue after the most gentle exercise, the sensation of weariness and aching in the limbs as from over-exertion when the patient is at rest, show clearly a depression of nervous energy that can be simulated by no known disease, but closely resembles the decrepitude of old age—that period of life when those constituents are particularly *scarce* which, next to water, form the principal elements of the brain tissue. The absorption of the fibrinous deposits of old cicatrices, causing re-ulcerations; the petechial spots gradually merging into ecchymoses, and constituting dangerous hæmorrhages in the vascular parts most superficial; the vertigo, palpitations, and feeling of faintness, point directly to an insufficiency in the materials of the nutrient fluid of the body, and to the elements in which that inadequacy lies—viz: hæmatin and globulin and other protein substances. Be the deficiency where it may, it must be in one. It cannot be in all, as such a coincidence would be incompatible with existence.

Claude Bernard, in his experiments on the renal vessels, has clearly shown the intimate interchange that can take place in the animal economy between fibrine and albumen, and teaches us to place but little reliance on those cases where, the other albuminoid constituents being partially absent, fibrine was found in scorbutic blood to be slightly on the increase, more especially when those examinations were made after previous profuse hæmorrhages had occurred.

It is unnecessary for me to deduce further proofs from the symptoms of the disease to strengthen my argument—such as the paleness and the livid leaden hue of the complexion from a deficiency of blood corpuscles, the stiffness of the joints from the absence of their natural albuminoid secretion, or the muscular wasting and debility owing to an insufficient supply of one of its chief elements, and from one of which sintonine is exclusively supplied; the life-supporting current not being able to spare those requisite protein ingredients from the stream that in many cases is unable to keep the wheel of life in motion.

I shall now, therefore, briefly state my experience of the scorbutic and antiscorbutic properties of the food usually supplied on long voyages, and the inferences I am led to deduce from it, and the generally received treatment of scurvy.

To persons who are familiar with the treatment of scurvy only in print, it will seem strange when I assert, from extensive inquiry and practical experience, that few ships, even amongst the best regulated transports, pass through a voyage of more than four, or even three, consecutive months, without being visited by this loathsome pestilence. And the publicity of its presence in those instances of protracted voyages would be more generally known were the medical officers in charge not ashamed to admit such an occurrence, owing to the present *popular* belief that an outbreak of scurvy is impossible when strict attention is paid to cleanliness, ventilation, employment of the mind and body, and the free use of lime-juice, and other supposed antiscorbutics. No doubt *ennui*, climatic changes, moisture, uncleanliness, impure air, &c., will predispose to scurvy, as they do to other diseases; but as to the generally supposed antiscorbutic properties of preserved milk, meats, vegetables, lime-juice such as we find in general use, and other specifics, I do not entertain the slightest belief.

The scorbutic properties of salt meat are, I think, undeniable, and the received theory as to its action is, I believe, the solvent power its alkalinity possesses on the albuminous components of the human tissues. This theory has been found to be incorrect, and serves to support my statement in a wonderful *degree*. The salting of meat does not produce scurvy directly by the introduction of saline particles into the system, but indirectly by dissolving out and removing from meat all its protein materials, which remain behind in the brine; and if men on board ship, instead of soaking the meat in fresh, and when that is not procurable, in sea water, in order to get rid of the salt, could be induced to consume the brine in their soup, puddings, &c., they would, in my opinion, be making use of the best antiscorbutic that is at the surgeon's disposal. Owing to this theoretical error as to hyper-alkalinity of the blood, pickles, lime-juice, &c. are administered with a view to its neutralization. I have seen them issued daily for four months as a prophylactic measure, and they proved as useless in that respect as they did in the cure of the disease after it had made its appearance. And what was this owing to? Not to a deficiency of acidity, for that they possessed; but to the absence of their protein elements, which in their fresh state they contained in abundance, but in this case had undergone that eremacausis to which protein compounds, both animal and vegetable, are so prone. The process, also, to which vegetables are subjected in pickling at large pickle manufactories, in my opinion, renders them totally useless as antiscorbutics. In order that a large stock may be ready for use at all seasons, they are enveloped in brine, and its action on them, and the results, are similar to those on meat.

It is not necessary for me to mention Garrod's and other theories on scurvy, as they have all been clearly refuted. Vegetables containing potassa also contain protein compounds in greater abundance, and it is owing to these latter constituents that plants belonging to the natural orders cruciferæ and aurantiaceæ claim their antiscorbutic qualities. As to the facts elicited from the practical experience of the antiscorbutic properties of preserved meats, milk, and vegetables, it is useless for me to enlarge; every medical officer who has been in charge of a large body of men on a long voyage can testify to their inefficacy; and this undoubtedly is owing to the protracted boiling, meats, vegetables, &c. are subjected to in the process of preservation and after-cooking, their albuminous substances by such means being rendered totally inert.

The backwoodsman of Canada has to work hard, and subsist on boiled potatoes, biscuit, and salt meat, all not of the best description. The protein waste of the tissues of their frames is greater than the salt pork, the boiled potatoes, and the old gluten biscuit can supply, and scurvy in consequence is frequently rife among them. Their infallible cure for the disease is eating the potatoe raw instead of boiled. For a similar reason milk, preserved on Appert's principle, is almost totally useless. Children fed on it for some weeks actually loathe its taste; for the cause of this, and the method adopted for its primary and secondary preservation, see Hassall's "Food and its Adulterations."

There are many incidents connected with the early history of scurvy, and its occurrence on land, that I could produce to strengthen my argument, but that seems to me superfluous. I shall conclude these remarks by surmising that the only link now required in this chain of assertions to convert them into a reality is a thorough chemical investigation of the nervous, muscular and albuminoid tissues of those who have died of scurvy, for a deficiency of protein compounds, and an analysis of the urine of scorbutic patients for the absence of urea. I certainly could not detect urea in the urine of fourteen such cases (not complicated with Bright's disease), and little, if any, protein compounds in the articles of ship's diet specified.—*Lancet.*

